# Comulang: towards a collaborative e-learning system that supports student group modeling

**DOI:** 10.1186/2193-1801-2-387

**Published:** 2013-08-15

**Authors:** Christos Troussas, Maria Virvou, Efthimios Alepis

**Affiliations:** Department of Informatics, University of Piraeus, Piraeus, Greece

**Keywords:** Group modeling, User modeling, User clustering, Classification, Computer supported collaborative learning, Machine learning

## Abstract

This paper describes an e-learning system that is expected to further enhance the educational process in computer-based tutoring systems by incorporating collaboration between students and work in groups. The resulting system is called “Comulang” while as a test bed for its effectiveness a multiple language learning system is used. Collaboration is supported by a user modeling module that is responsible for the initial creation of student clusters, where, as a next step, working groups of students are created. A machine learning clustering algorithm works towards group formatting, so that co-operations between students from different clusters are attained. One of the resulting system’s basic aims is to provide efficient student groups whose limitations and capabilities are well balanced.

## Introduction

In recent years, the rapid development of high and new technology has opened new horizons in computer-assisted instruction. Intelligent Tutoring Systems are based on computer models of instructional content and support the learning, by providing personalized instruction to students. In this way, students may learn one or more foreign languages. European reality necessitates multiple language learning (European Union), so the students may further benefit from this educational process. For this reason, the need of systems that incorporate intelligence is even greater when students are taught more than one foreign language simultaneously (Virvou & Troussas 2011).

Moreover, our world has witnessed major improvements in the areas of transportation and telecommunications. These important changes have permitted the rise of the phenomenon of globalization by which regional economies, societies, and cultures have become integrated through a global network of people. As a result, all the emerging needs of modern life accentuate the importance of learning foreign languages (Kurata 2010). Considering the scientific area of Intelligent Tutoring Systems (ITSs), there is an increasing interest in the use of computer-assisted foreign language instruction (Virvou & Troussas 2011). In this way, students may learn a foreign language, by using a computer-assisted application. Especially, when these systems offer the possibility of multiple-language learning at the same time, the students may further benefit from this educational process (Virvou et al. 2000). The need for tutoring systems that may provide user interface friendliness and also individualized support to errors via a student model are even greater when students are taught more than one foreign languages simultaneously (Virvou & Troussas 2011). A solution to this problem may be the integration of the technology of Intelligent Tutoring Systems (ITSs), so as to provide adaptive tutoring to individual students. ITSs offer intelligence and adaptivity to individual students’ needs, via student modeling. The individual student model for each student contains information about the knowledge level and the error handling of the student in each concept of multiple language learning. Hence, error diagnosis is a module which supports the students while studying theory and solving exercises (Tsiriga & Virvou 2004). Socialization has important pedagogical implications in collaborative learning that support the learners’ personal relationships and social interaction with their classmates (Caballé et al. 2010). Therefore, the support of collaboration in multiple language learning may promote the learning process. When adaptive personalized e-learning systems could accelerate the learning process by revealing the strengths and weaknesses of each student in a collaborative environment, they could dynamically plan lessons and personalize the communication and didactic strategy (Licchelli et al. 2004). Machine learning techniques can be used for acquiring models of individual users interacting with educational systems and group them into communities or stereotypes with common interests (Papatheodorou 2001), so that the student reap the benefits of collaboration.

In view of the above, we have implemented an educational system which promotes learning through working in teams and sets the standards needed in order to promote educationally beneficial instruction to students in a multi-language learning environment. The collaboration between students from groups of different linguistic competences facilitates the contextualized communicative nature of multiple language learning. The procedure of grouping is conducted by the incorporation of machine learning techniques for clustering. Building a student model involves defining crucial matters such as the degree of specialization of the students that are modeled, their knowledge and capabilities and also the way of giving assistance, providing feedback and interpreting the behavior of the learner.

This paper is organized as follows. In section 1, we present related scientific work. Following, in sections 2 and 3, we discuss our system’s architecture and we give an overview of the resulting system. Finally, in section 4, we come up with a discussion about the usability of the resulting system and we present our future plans.

## 1. Related work

Error handling is a quite significant issue in educational software. In all aspects of Intelligent Tutoring Systems, either incorporating collaborative support or machine learning techniques, students’ error handling plays an important role. In this section, we try to imprint the speckle of the scientific progress in computer supported collaborative learning and machine learning in terms of error handling.

### 1.1. Computer-supported collaborative learning

In (Kessler & Bikowkski 2010), the authors reported on attention to meaning among teachers as they collaboratively constructed a wiki in an online course and focus on the nature of individual and group behavior when attending to meaning in a long-term wiki-based collaborative activity as well as the student’s collaborative autonomous language learning abilities. In (Ada 2010), the authors described a pedagogical design that creates collaborative opportunities and experiences to promote critical reflection to learners. The authors of (Caballé et al. 2010), attempted to bridge relevant aspects of technology in support for collaborative learning and provide a tighter view by means of a multidimensional approach. In (Baghaei et al. 2006), the authors has introduced a constraint-based ITS, that supports both problem solving and collaborative learning, for the learning of object-oriented design using Unified Modeling Languages. In (Guerrero et al. 2010), the authors designed a collaborative activity and a software tool to support teaching grammar to primary education by creating interdependencies among students. Reference (Read et al. 2005) presented a system that helps students to improve their linguistic production combining individual and collaborative activities in a constructivist methodology with a way to overcome technological language analysis difficulties. In (Cohen & Scardamalia 1998), the authors proposed a wiki-based tool for collaborative writing in language learning. They also collected data for the analysis and evaluation of collaborative autonomous learning.

### 1.2. Computer-assisted language learning

AutoTutor is a CALL system, developed by (Graesser et al. 2005), which simulates a human tutor by promoting the conversation and provides feedback to the learner, pumps him/her for more information, gives hints, fills missing information with assertions, identifies and corrects bad answers, answers learner’s questions and summarizes answers. Another CALL system is rEcho, which is developed by (Zhou et al. 2007). It can give relevant feedback through articulatory animation and error trends grouping. SignMT was implemented by (Ditcharoen et al. 2010) to translate sentences and phrases from different sources in four steps, which are word transformation, word constraint, word addiction and word ordering. Another computer-based program on second language acquisition is Diglot Reader, which was developed by (Christensen et al. 2007) and is used in a way that students may read a native language text with second language vocabulary and grammatical structures increasingly embedded within the text. VIRGE, developed by (Katsionis & Virvou 2008), works as an adventure virtual reality game but it has educational content as well and supports personalized learning based on a student modeling component.

Furthermore, in (Antal & Koncz 2011), the authors reviewed the student modeling problem for computer-based test systems and also proposed a novel method for the graphical representation of student knowledge. In (Savvopoulos & Virvou 2010), the authors presented an intelligent tutoring component which helps elderly people in an adaptive way and predicts their mistakes. Moreover, in (Ferreira & Atkinson 2009), the authors presented a model of corrective feedback for an ITS for Spanish as a foreign language and proposed the design of a component of effective teaching strategies into this ITS. In (Dickinson et al. 2008), the authors designed a paper-based system that provides feedback on particle usage for first-year Korean learners, who learn a second language. TAGARELA is an individualized instruction program, implemented by (Amaral & Meurers 2007), which analyzes student input for different activities, provides individual feedback, motivates a broader perspective of student models for Intelligent Computer-Assisted Language Learning and incorporates insights from current research on second language acquisition and language testing. In (Tsiriga & Virvou 2004), the authors presented a framework for the initialization of student models in web-based educational applications.

Moreover, in (Virvou et al. 2000), the authors presented a novel approach for the evaluation of ITSs, which relies on an agent that may be used as a simulated student-user and incorporates modeling techniques. Finally, in (Virvou & Troussas 2011), the authors described a ubiquitous e-learning tutoring system for multiple language learning, called CAMELL (Computer-Assisted Multilingual E-Language Learning). It is a post-desktop model of human-computer interaction in which students “naturally” interact with the system in order to get used to electronically supported computer-based learning. Their system presents advances in user modeling, error proneness and user interface design.

### 1.3. Machine learning

In (Basile et al. 2011), the authors proposed the exploitation of machine learning techniques to improve and adapt the set of user model stereotypes by making use of user log interactions with the system. To this end, a clustering technique is exploited to create a set of user models prototypes; then, an induction module is run on these aggregated classes in order to improve a set of rules aimed as classifying new and unseen users. Their approach exploited the knowledge extracted by the analysis of log interaction data without requiring an explicit feedback from the user. In (Niño 2009), the authors presented a snapshot of what has been investigated in terms of the relationship between machine translation (MT) and foreign language (FL) teaching and learning. Moreover, the author outlined some of the implications of the use of MT and of free online MT for FL learning. In (Friaz-Martinez et al. 2007), the authors investigated which human factors are responsible for the behavior and the stereotypes of digital libraries users so that these human factors can be justified to be considered for personalization. To achieve this aim, the authors have studied if there is a statistical significance between the stereotypes created by robust clustering and each human factor, including cognitive styles, levels of expertise and gender differences. In (Virvou & Chrysafiadi 2006), the authors described a web-based educational application for individualized instruction on the domain of programming and algorithms. Their system incorporates a user model, which relies on stereotypes, the determination of which is based on the knowledge level of the learner. In (Licchelli et al. 2004), the authors focused on machine learning approaches for inducing student profiles, based on Inductive Logic Programming and on methods using numeric algorithms, to be exploited in this environment. Moreover, an experimental session has been carried out from the authors, comparing the effectiveness of these methods along with an evaluation of their efficiency in order to decide how to best exploit them in the induction of student profiles. In (Tsiriga & Virvou 2004), the authors introduced the ISM framework for the initialization of the student model in Web-based ITSs, which is a methodology that uses an innovative combination of stereotypes and the distance weighted k-nearest neighbor algorithm to set initial values for all aspects of the student model. In (Webb et al. 2001), the authors explained that user modeling poses a number of challenges for machine learning that have hindered its application in user modeling, including the need for large data sets, the need for labeled data, the concept drift and computational complexity. In (Beck & Park 2000), the authors constructed a learning agent that models student behavior at a high level of granularity for mathematics tutor, by using traces from previous users of the tutor to train the machine learning agent.

However, after a thorough investigation in the related scientific literature, we came up with the result that there was no implementation of multilingual educational systems that offer error diagnosis in computer-supported collaborative multiple language learning using student clustering. Hence, we implemented a prototype system, which incorporates intelligence in its diagnostic component and offers the possibility of collaboration to students according to their knowledge level, as it is imprinted on the clustering conducted by the k-means algorithm.

## 2. General architecture of the system

Our system is called “Comulang”, given that it supports collaboration in a multiple language learning platform. The architecture of Comulang follows the basic line of ITS architectures. It is widely agreed that the major functional components of an ITS architecture are the domain knowledge, the student modeler, the advice generator and the user interface (Hartley & Sleeman 1973); (Burton & Brown 1976); (Wenger 1987). In this section we will briefly describe the domain knowledge, the advice generator and the user interface, while the student modeling is described in detail subsequently.

The domain knowledge of the system consists of procedures and rules about prerequisite grammatical concepts along with the evaluation part of each student. The domain knowledge of our system is responsible for performing the following tasks:

- Parsing the exercise sentence
- Identifying string similarities by matching a student’s given “exact” wrong answer with the systems correct stored answer
- Identifying string meaning similarities between the given and the correct answer by translating these two answers to the system’s available supported languages
- Identifying the semantic relationships between the subject, the verb and the object and judging whether the exercise sentence makes sense
- Conjugating the verb into the appropriate tense in the passive/active sentence

The tasks performed by our domain knowledge are supported by a knowledge base that represents the proper use of verbs and articles and pronouns in the exercise sentences. For each word, a number of attributes are associated with it. For example, in the case of a verb one attribute represents whether it is used as main verb or not, whether it is irregular and other attributes concern the Greek translation of the verb and translations to other languages, such as French. In addition, the words included in the vocabulary are related through a semantic net, so that the system may be able to identify whether a sentence makes sense or not.

The advice generator is responsible for acting when the user makes an error. In such case, it tries to respond in the most appropriate way by informing the user about what the cause of the error has been and by showing him/her the relevant part of the theory. For example, if a student made a mistake that is related to the use of verbs then the advice generator will indicate the student the part of the theory that deals with this subject. Furthermore, the advice generator is activated in the theoretical section where the student learns the theory. In this way, the student is assisted in order not to be confused when learning the theory in the two different foreign languages. Moreover, the advice generator is the component that is responsible for constructing new fill-in-the-gap-exercises, as well as showing to the learner a set of grammar pages on demand.

Finally, the user interface is quite important for this kind of application, because it can stimulate the student’s interest in learning multiple languages. In addition, it conveys the functionality of a computer application to the user and translates the user’s input into a machine-specific format (Plass 1998). The user interface of our system is a multimedia user interface, which involves all the necessary elements (such as clarity, familiarity, responsiveness, efficiency, consistency) so that it can attract the student’s interest in the subject.

### 2.1. Modeling the student knowledge

The user modeler is responsible for preserving the system’s estimation of the learner’s proficiency in the domain as well as his/her proneness to commit errors. In addition, it adapts the behavior of the system directly to the needs of the learner. The emphasis on the student modeling component has been placed on the bi-directional interaction of two sub-components: the long term and the short term student model.

The system constructs a student model, which serves as a source of information that can be used for the interpretation of the student’s actions and possible mistakes in solving exercises. The student modeler checks the student’s answer against the expert’s answer and in a case of an error, it performs error diagnosis (Virvou & Troussas 2011). While performing error diagnosis, the student’s answer is checked against the set of the erroneous versions that the system is able to identify. One important source of errors is considered to be the confusion between the two languages taught. Error diagnosis is performed by the short term student model.

Another responsibility of the student modeler is to form the long term student model. The long term student model constitutes a history model of the student’s weaknesses and progress. The long term student model influences the process of error diagnosis. For example, if a student has been recorded to have frequently made accidental slips but no grammar errors at all, then in case of ambiguity the former cause is favored. In addition, students have the possibility of checking their own profiles, which provide information about their knowledge level and progress, and therefore benefit from viewing their own student models. Hence, this kind of information is used not only for refining the error diagnosis process but also for presenting it to the user.

### 2.2. Error diagnosis

In a past empirical study, conducted by the authors, among human teachers and their students, we have showed that both teachers and students were very interested in knowing which categories of errors individual students were prone to and which grammatical concepts they had mastered. This result was in accordance with results from other empirical studies as well. Human tutors also considered it important that the type of exercises should be similar to the type used in exam papers of state schools. Therefore, the student, while working with the system, is given three types of exercise to select from (Virvou et al. 2000):

- Multiple choice exercises: Multiple choice questions are the most widely used as they are a mainstay of achieving testing and also provided us with the ability to measure the students’ achievements. Multiple choice is a form of assessment in which the user is asked to select the best possible answer out of the choices from a list. One of them is the correct, while the others answers are erroneous. Furthermore, in our system, there has been a significant effort for the analysis of possible students’ mistakes in the design of the diagnostic component of the student modeler. Namely, we have developed a bug library, which keeps all the erroneous answers and correlates them with a category of error, so that the student should have an integral idea of his/her knowledge level and know exactly where s/he has weaknesses in. Each erroneous student’s answer is checked against the set of the erroneous versions that the system stores in its bug library.
- Exercises where the user is asked to fill in the gaps inside a sentence: In this case, there are twenty questions, which are different for each student and based on his/her individualized user model. Specifically, while filling in the gaps, the system requests input from the student modeling component, as to what the main difficulties of the particular student are. In this way, the selected question is relevant to those parts of the theory that the student has been recorded to have weaknesses in. After completing the gaps, the system gives the results of the final examination. It shows the grade of the student and spots the erroneous answers. Furthermore, the system corresponds to the erroneous answers for each category of errors stored in the bug library.

Error diagnosis is performed by the system in the “Solving Exercises Mode”. In multiple choice exercises error diagnosis is quite simple. For every erroneous answer that the student may select, there is an associated misconception. Therefore, depending on the erroneous selection that the student has made, a corresponding error message is presented, explaining the cause of the mistake.

In the case of exercises where the student is asked to fill in the gaps in a sentence, error diagnosis becomes more sophisticated since in this case the student is allowed to be more creative than in multiple choice exercises. In the “rewrite” type of exercise the student is given a sentence in one voice (active or passive) and is asked to rewrite the sentence using another voice. The system incorporates knowledge about how to convert a sentence from one voice to another correctly. However, if the student’s answer differs from the system’s expectation then the system performs error diagnosis.

The cases where the system performs error diagnosis result from the following steps of a parsing algorithm:

- The system counts the words that constitute the sentence that the student has given as an answer. This sentence could be either in the active or in the passive voice. If the number of the words in the student’s answer is different from the ones that the system expects, then error diagnosis is performed.
- In order to successfully recognize one or more of the categories of errors, our system incorporates two algorithmic approaches. The first algorithm tries to find string similarities by matching a student’s given “exact” wrong answer with the systems correct stored answer. If string matching occurs in a high percentage the system decides whether the mistake lies between the following categories: article and pronoun mistakes, spelling mistakes, verb mistakes and unanswered questions.
- Correspondingly, using the second algorithm, the system also tries to find meaning similarities between the given and the correct answer by translating these two answers to the system’s available supported languages. As an example, the student may have used “We are” instead of “Nous sommes”, which is the French equivalent.

Error diagnosis is performed by matching each identified error against the buggy knowledge and corresponding explanations of the system’s knowledge base. In cases where more than one category of error and/or explanations match the identified mistake, then ambiguity resolution is performed, using the priorities of the categories of errors and the long term student model (Virvou et al. 2000).

### 2.3. Student groups as clusters

As mentioned before, user models contain personal information about the user, such as his/her knowledge level, progress, age, occupation, emotional state etc. These type of information are not directly used as a means of adaptation of the system to the user, but can be used in order to categorize the user into a stereotype, which in turn allow the system to anticipate some of the user’s behavior (Papatheodorou 2001). Stereotypes are used to organize the users of our system, in terms of common behavior, into meaningful groups. The clustering of users into groups with common interests is very useful in learning multiple languages and for this reason this process is conducted by the incorporation of an unsupervised clustering algorithm, namely the K-means algorithm.

K-means clustering algorithm is one of the simplest unsupervised learning algorithms that solve the well known clustering problem. The procedure follows a simple and easy way to classify a given data set through a certain number of clusters (assume k clusters) fixed a priori. The main idea is to define k centroids, one for each cluster. These centroids should be placed in a cunning way because of different location causes different result. So, the better choice is to place them as much as possible far away from each other. The next step is to take each point belonging to a given data set and associate it to the nearest centroid. When no point is pending, the first step is completed and an early “grouping” is done. At this point we need to re-calculate k new centroids as barycenters of the clusters resulting from the previous step. After we have these k new centroids, a new binding has to be done between the same data set points and the nearest new centroid. A loop has been generated. As a result of this loop we may notice that the k centroids change their location step by step until no more changes are done. In other words centroids do not move any more. Finally, this algorithm aims at minimizing an objective function, in this case a squared error function. The objective function

where  is a chosen distance measure between a data point  and the cluster centre *c*_*j*_, is an indicator of the distance of the n data points from their respective cluster centers.

The algorithm is composed of the following steps:

- Place K points into the space represented by the objects that are being clustered. These points represent initial group centroids.
- Assign each object to the group that has the closest centroid.
- When all objects have been assigned, recalculate the positions of the K centroids.
- Repeat Steps 2 and 3 until the centroids no longer move. This produces a separation of the objects into groups from which the metric to be minimized can be calculated.

### 2.4. Implementation of k-means algorithm

From empirical studies, we have n sample feature vectors x1, x2, …, xn all from the same class, and we know that they fall into k compact clusters, k < n. There are several categories of methods for deciding the number of clusters k. One simple principle that we incorporated in the implementation of k-means algorithm sets the number to (Mardia et al. 1979):

Let x1, x2, …, xn be the vectors of students’ characteristics, namely their knowledge level and age, since they have been found to be quite significant in past language learning applications Tsiriga & Virvou (2004). Let mi be the mean of the vectors in cluster i. If the clusters are well separated, we can use a minimum-distance classifier to separate them. That is, we can say that x is in cluster i if || x - mi || is the minimum of all the k distances. This suggests the following procedure for finding the k means:

- Make initial guesses for the means m1, m2, …, mk
- Until there are no changes in any mean
- Use the estimated means to classify the samples into clusters
- For i from 1 to k
- Replace mi with the mean of all of the samples for cluster i
- end_for
- end_until

**Figure 1:**
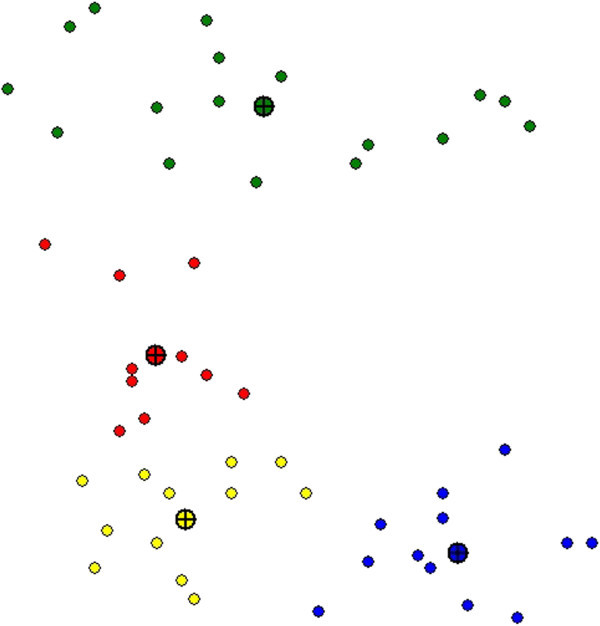
**Snapshot of student clustering.**

Figure 1 is an example showing how the means m1, m2, m3 and m4 move into the centers of the clusters.

For the incorporation of the machine learning approach into the resulting mobile multilingual system Virvou et al. (2012), we make the following basic steps:

- For the initialization of the system the algorithmic techniques receive as input, pre-stored data or data from empirical studies. In our system we have used two fundamental characteristics which in accordance with the authors’ expertise in the domain tend to influence the educational procedure:
  - the age of students and
  - their level of knowledge in one of the foreign language taught.
  These characteristics have been found quite significant in past language learning applications (Tsiriga & Virvou 2004).
- Machine learning techniques are used as a next step in order to describe efficiently the circumstances that underlie the student’s actions in terms of their behavioral patterns and preferences.
- Based on the aforementioned characteristics, the system creates clusters of the already existing students. These clusters contain valuable information about their members, considering their behavior, their preferences and generally their interaction with the system.

### 2.5. Collaboration supported by clustering

K-means algorithm has created four discrete clusters. Each observation belongs to the cluster with the nearest mean. Every cluster has specific characteristics and the students, who belong to a cluster, become communicants of these characteristics.

**Figure 2:**
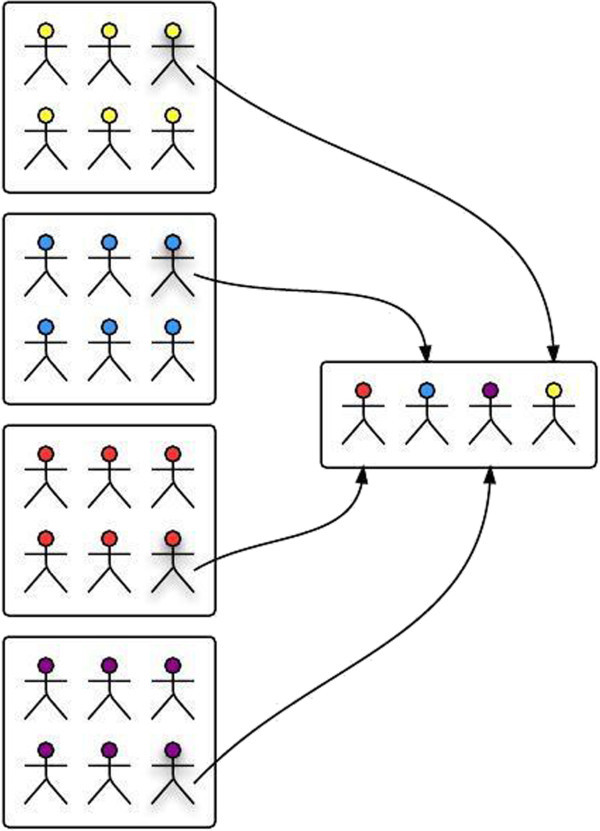
**Illustration of collaboration among student groups.**

In case of a problem or question, the student has the possibility of collaborating with his/her peers by asking for their assistance. Then, the system sophisticatedly picks a student from another cluster in a way that s/he can help the student who asked for help through collaboration, as illustrated in Figure 2. The selection of the student, who is going to provide and share knowledge to his/her peers, is conducted based on the long term student models. In particular, the students have the possibility to cooperate in order to master each one of the two languages, by sharing their knowledge of different background level. For example, when a student conducts knowledge errors or accidental slips, s/he will be proposed by the system to collaborate with a student from another cluster who avoids knowledge and accidental slips and can be helpful to their peers. Our system relies on the interdependencies among group members, such as the need for information interchange and the need for explicit knowledge sharing.

## 3. General overview of Comulang

**Figure 3:**
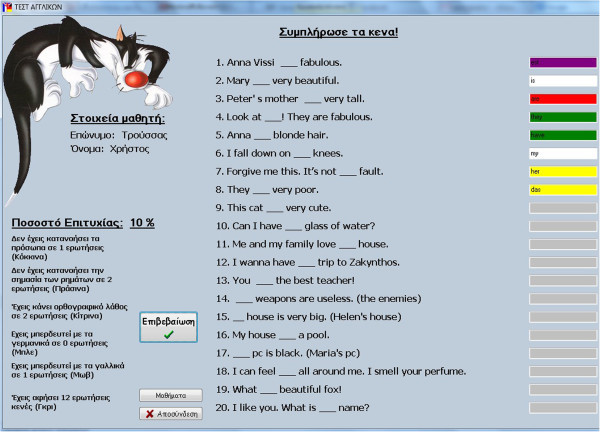
**Student’s errors.**

**Figure 4:**
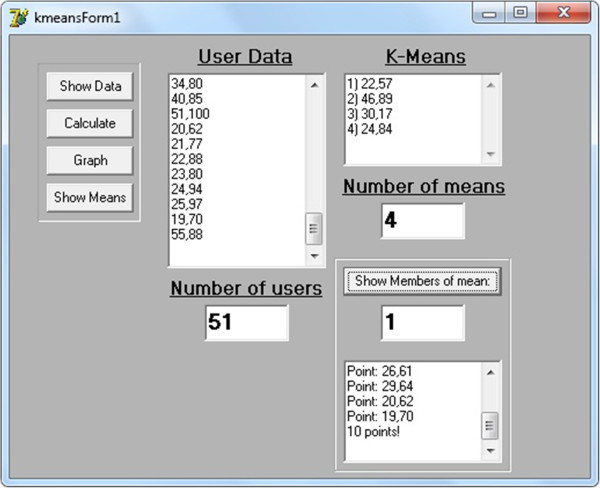
**Snapshot of k-means clustering algorithm.**

Figure 3 illustrate a snapshot of the operating educational application, where a student is completing a “fill-in-the-gap” exercise and taking the system’s feedback. More specifically, it illustrates a categorization to a student’s specific errors. The student can be evaluated and check where s/he is wrong and what type of mistake s/he has made. The different colors indicate different type of errors, such as errors in articles or pronouns, verb mistake, spelling mistakes, confusion with the German or French language or unanswered questions. Finally, Figure 4 illustrates a report of k-means, the initial user data and the resulting k-mean vectors.

## 4. Conclusions, discussion and future work

In this paper, we have incorporated collaboration among learners in a computer assisted multiple language learning environment supported by student grouping. Effective collaborative learning consists of students perceiving the importance of working actively with their peers in order to learn and act in ways which ameliorate the educational procedure and accentuates the value of cooperation. Individual learning is also promoted along with the learning of the entire group of students.

Furthermore, error diagnosis techniques are incorporated in our computer assisted collaborative multiple language learning environment supported by student grouping. In our multi-language environment, it is quite important to encourage flexibility in grading collaborative work by proposing co-operations based on individual models of students who belong in different groups emanating from clustering algorithmic techniques.

The resulting system, Comulang, is already presented to a small group of tutors and to a larger group of students. Both have found its underlying architecture very interesting and promising towards the creation of successful distance learning courses. However, it is quite obvious that a system that incorporates working through groups needs to be evaluated for a long period of time, for its group members to interact with each other sufficiently.

To this end, it is in our next future plans to long term evaluate our system in order to examine the degree of its usefulness in multiple language learning environments. Moreover, we are going to evaluate the usefulness of collaboration among students of different clusters. The authors of this paper strongly believe that the verification of this attempt through a scholastic evaluation will open the way for the incorporation of their approach into computer-based educational systems in general.
